# Where Do We Stand for Nerve Regeneration and Functional Recovery After Vascularized Composite Allotransplantation?

**DOI:** 10.1002/micr.70192

**Published:** 2026-02-10

**Authors:** Olivier Mathieu, Leonard Knoedler, Thomas Schaschinger, Jakob Fenske, Felix J. Klimitz, Maxime Jeljeli, Curtis L. Cetrulo, Alexandre G. Lellouch

**Affiliations:** ^1^ Division of Plastic and Reconstructive Surgery Cedars‐Sinai Medical Center Los Angeles California USA; ^2^ Charité – Universitätsmedizin Berlin, Corporate Member of Freie Universität Berlin and Humboldt‐Universität zu Berlin Department of Oral and Maxillofacial Surgery, Augustenburger Platz 1, 13353 Berlin Germany; ^3^ Division of Plastic Surgery, Department of Surgery Yale New Haven Hospital, Yale School of Medicine New Haven Connecticut USA; ^4^ Vascularized Composite Allotransplantation Laboratory, Center for Transplantation Sciences Massachusetts General Hospital, Harvard Medical School Boston Massachusetts USA; ^5^ Université Paris Cité, Inserm The Paris Cardiovascular Research Center, Team Endotheliopathy and Hemostasis Disorders Paris France; ^6^ Hematology Department AP‐HP, Hôpital Européen Georges Pompidou Paris France

**Keywords:** face transplantation, functional recovery, nerve regeneration, vascularized composite allotransplantation, VCA

## Abstract

**Introduction:**

Vascularized composite allotransplantation (VCA) offers a reconstructive solution for patients with severe limb and facial defects by transplanting multiple tissue types—including skin, muscle, bone, and nerves—as a single unit. This review examines current challenges in nerve regeneration and functional recovery following VCA, and assesses emerging strategies aimed at overcoming these hurdles.

**Methods:**

A comprehensive literature review was conducted, focusing on clinical outcomes from upper limb and facial transplantation programs worldwide. Studies evaluating rehabilitation protocols, transplantation levels, follow‐up durations, and experimental strategies (cellular therapies, biomaterials, and neurotrophic factor delivery) were analyzed to determine their impact on nerve regeneration and functional recovery.

**Results:**

Clinical evidence indicates that while protective sensation can return within months after VCA, motor function recovery often extends over several years. Variability in outcomes has been noted, largely due to differences in rehabilitation practices, assessment metrics, and follow‐up duration. Experimental approaches, including stem cell‐based therapies—especially those using adipose‐derived stem cells—demonstrate potential for enhancing axonal regeneration and modulating immune responses, although the long‐term benefits and standardized measures remain to be fully established.

**Conclusions:**

Despite promising advancements in surgical techniques and immunosuppressive regimens, effective nerve regeneration in VCA remains a significant challenge. The variability in clinical outcomes underscores the need for standardized functional assessment protocols and further research into novel regenerative therapies. Future studies should focus on refining these therapeutic strategies to improve long‐term functional recovery and minimize the reliance on chronic immunosuppression.

## 1 Introduction

Vascularized Composite Allotransplantation (VCA) has become a promising treatment option for reconstructing limbs and facial structures in patients with severe tissue defects (Thuong et al. 2019). Unlike traditional organ transplants, VCA involves the transfer of multiple tissue types—skin, muscle, bone, and nerves—as a single functional unit. Achieving functional recovery post‐VCA depends on both effective immunosuppression and successful peripheral nerve regeneration to restore motor and sensory functions (Leto Barone et al. 2015). Nerve tissue is particularly vulnerable to chronic immune‐mediated damage, which can impair regeneration and functional recovery (Tang et al. 2022).

Advancements in surgical methods and immunosuppressive therapies have led to notable improvements in VCA outcomes (Knoedler, Schaschinger, et al. 2025). However, nerve regeneration remains a bottleneck, often limiting full restoration of motor and sensory capabilities (Tung and Mackinnon 2018). Nerve repair in VCA differs from conventional peripheral nerve surgery: regeneration occurs within an allogeneic and highly immunogenic environment, under the influence of long‐term immunosuppression, often across long regeneration distances and within nerves of mixed sensory–motor composition. These factors can delay or compromise functional recovery despite the use of techniques known to enhance regeneration in standard nerve repair. Both preclinical and clinical studies have shown that stem cells, neurotrophic factors, and biocompatible materials can enhance regeneration and improve outcomes. However, few studies have synthesized existing evidence (Kostereva, Wang, Fletcher, Unadkat, et al. 2016).

This research gap leaves untapped potential to improve outcomes following VCA surgery. This review aims to provide a comprehensive overview of current strategies and future directions to enhance nerve regeneration and functional recovery following VCA. We will examine the biological mechanisms involved, explore relevant animal models, discuss clinical findings, and highlight future prospects in this rapidly advancing field.

## 2 Methods

A comprehensive literature review was conducted using PubMed and Scopus databases up to March 2025. Search terms included “vascularized composite allotransplantation,” “nerve regeneration,” “functional recovery,” “hand transplantation,” and “face transplantation.” Both clinical and preclinical studies addressing peripheral nerve regeneration, immunosuppressive effects, and rehabilitation strategies in VCA were included. Reference lists of key articles were manually screened to identify additional relevant studies. Data were extracted on mechanisms of regeneration, sensory and motor recovery outcomes, and experimental interventions aimed at enhancing regeneration.

## 3 Results

### 3.1 Mechanisms of Nerve Regeneration

Nerve regeneration following VCA involves several mechanisms, beginning with Wallerian degeneration, which creates an environment for axonal regrowth (Moore et al. 2009). Schwann cells play a crucial role by forming bands of Büngner and releasing neurotrophic factors like nerve growth factor (NGF) and brain‐derived neurotrophic factor (BDNF), which support axonal growth and neuronal survival. Host Schwann cells infiltrate the nerve allograft, enhancing axonal regeneration and reducing the need for immunosuppression (Fornaro et al. 2001; Fukaya et al. 2003; Jensen et al. 2005; Tseng et al. 2003).

The regeneration of nerves in VCA differs from that in autografts due to allogeneic tissues and the need for immunosuppression (Moore et al. 2009). As with autografts, the success of nerve regeneration in VCAs is influenced by the distance over which nerves must regenerate. Longer distances can pose significant challenges as the regenerating axons may fail to reach their targets. This is particularly relevant in proximal limb transplants, where the nerves must traverse greater lengths to reinnervate distal muscles and sensory receptors (Tung and Mackinnon 2018). Overall, nerve regeneration in VCA is a multifaceted process that relies on the coordinated efforts of Schwann cells, neurotrophic factors, and the careful management of the immune response. Understanding these mechanisms is essential for developing strategies to enhance nerve regeneration and improve functional outcomes in VCA patients.

### 3.2 Nerve Regeneration in Human VCA Recipients

Clinical outcomes of VCA in humans demonstrate varying degrees of functional recovery, with significant progress in restoring sensory and motor functions. Protective sensation, such as pain and temperature perception, typically returns within the first few months post‐transplantation, while discriminative sensation and motor function may take several months to years to fully recover (Shores et al. 2017). Functional outcomes following upper extremity transplantation have been highly encouraging, with many patients achieving significant improvements in sensation, motor function, and independence in daily activities (Thuong et al. 2019). However, variability in reporting and data collection persists due to several factors, including differences in follow‐up durations, assessment methods, and transplantation levels. Longer follow‐up periods are commonly associated with better functional outcomes as recovery continues to progress over multiple years.

Additionally, the lack of a gold standard for functional outcome assessment has led to inconsistencies in data acquisition (Landin et al. 2012).

The French experience (Lyon, France), reported in 2015, highlighted outcomes in five bilateral hand transplant recipients with follow‐up periods ranging from 4 to 13 years (Bernardon et al. 2015). All patients developed at least grade S3 sensation (i.e., a classification on the modified Highet scale assessing sensory and motor recovery in peripheral nerve injuries where S0 represents complete absence of sensation and S4 represents normal or near‐normal sensation) (Dellon et al. 1974) and achieved grip strength corresponding to 4%–28% of normal function. Carroll Test scores (Carroll 1965) averaged 69% (58%–88%) for dominant hands and 55% (39%–81%) for non‐dominant hands, with an average DASH score (Jester et al. 2005) of 15 (range 4–42).

The Louisville team reported functional data in 2011 for six of their 10 hand transplant recipients, including one bilateral case (Kaufman and Breidenbach 2011). Protective sensation was restored in all recipients, with the first patient achieving near‐normal two‐point discrimination (5–9 mm) and grade 4/5 thumb intrinsic function. Carroll scores ranged from 57 to 69/99, indicating good functional outcomes. One graft loss occurred due to severe intimal hyperplasia, but all other recipients regained independence in daily living. The Spanish experience, published in 2011, described outcomes in three bilateral hand transplant recipients (Cavadas, Landin, et al. 2011). All patients showed improved Chen scores (i.e., a standardized scale for evaluating functional recovery after microsurgical reconstructions) and reduced DASH scores post‐transplantation, with HTSS scores (Lanzetta and Petruzzo 2007) ranging from 73.5 to 79.5. Additional surgical interventions were performed to enhance function and aesthetics (Cavadas, Ibañez, and Thione 2011). The Innsbruck team reviewed outcomes for four transplant recipients, including bilateral hand, forearm, and wrist transplants (Hautz et al. 2011). Protective and discriminative sensation was restored in all cases, with HTSS scores rated as good or excellent. DASH scores varied widely (5%–62.5%), but unilateral and bilateral recipients showed similar rehabilitation progress and patient satisfaction.

The Polish experience, reported in 2011, included seven upper limb transplants in six patients, with two performed at more proximal levels (mid‐humerus and distal humerus) (Jabłecki 2011; Kaczmarzyk 2012). All patients regained protective sensation, with two achieving two‐point discrimination. One graft loss occurred due to surgical complications, including thrombosis of arterial collaterals (Jabłecki 2011).

The joint program between Johns Hopkins University and the University of Pittsburgh reported outcomes for seven patients (four bilateral and three unilateral transplants) (Schneeberger et al. 2013; Shores et al. 2013). Carroll scores ranged from 24 to 70 for dominant hands and 18 to 64 for non‐dominant hands. Two unilateral recipients experienced graft loss due to non‐compliance with immunosuppression, but the majority showed significant functional improvement, with DASH scores decreasing over time. Bilateral recipients demonstrated the largest magnitude of improvement, with some achieving full independence.

According to the International Registry on Hand and Composite Tissue Transplantation (IRHCTT), the majority of hand‐grafted patients developed protective sensibility: 90.0% of them regained tactile sensibility and 82.3% also a discriminative sensibility (Petruzzo and Dubernard 2011). Motor recovery allowed patients to regain the ability to perform most activities of daily living (Petruzzo and Dubernard 2011). Distal transplants consistently outperformed proximal ones in sensory and motor recovery, though proximal and bilateral transplants showed the greatest functional improvement over time. While long‐term data on the world's first bilateral arm transplantation is not available to the scientific community yet, personal communication revealed that the patient showed rapid and sustainable sensory and motor recovery (personal mail communication, Prof. Dr. Machens, 03.09.2025).

There is limited objective data in the literature regarding sensory and motor recovery following facial transplantation. The 15 face‐grafted patients described in the IRHCTT showed improvements in eating, drinking, and speaking, enabling them to lead a normal social life (Petruzzo and Dubernard 2011; Knoedler, Hoch, et al. 2025). In a study conducted by Siemionow et al. (2009), 6 months after a facial transplantation, sensory discrimination returned across the entire facial skin. Two‐point discrimination, measured using a standardized pressure‐specific sensory device, was observed in areas under the lower eyelids, the upper lip, and the tip of the nose on both sides of the graft. Motor recovery, assessed through repose, pucker, smile, and pronunciation of vowels, showed gradual improvement over the six‐month follow‐up. Advances were noted in facial mimetics, including symmetric smiling and upper lip occlusion. However, movements of the upper lip and lower eyelid remained incomplete. Functional restoration of key oral and nasal functions was also documented. This patient regained the ability to consume solid foods independently, eliminating the need for gastric‐tube feeding. The restoration of the upper lip enabled drinking from a cup.

The variability in functional outcomes among patients remains a major limitation, with some achieving near‐complete recovery while others experience only partial restoration of function. This variability is often attributed to differences in nerve regeneration capacity, the timing and quality of rehabilitation, and individual patient factors such as age and comorbidities. Furthermore, the lack of standardized protocols for immunosuppression and rehabilitation complicates the comparison of outcomes across studies, reinforcing the need for more rigorous clinical trials and long‐term follow‐up data.

### 3.3 Impact of Acute and Chronic Rejection on Nerve Regeneration in VCA

Both acute and chronic rejection can adversely influence nerve regeneration after VCA, albeit through distinct mechanisms and timelines. The relationship between acute rejection episodes and nerve regeneration in VCA has been insufficiently addressed in the literature, yet available experimental evidence suggests that rejection per se does not abolish axonal regrowth (Yan et al. 2013). In a rat orthotopic limb VCA model, a single early or late acute rejection episode, promptly reversed with intensified immunosuppression, did not significantly alter regenerating axon counts compared with fully immunosuppressed or syngeneic controls (Yan et al. 2013). However, late rejection was associated with reduced muscle force despite similar axon numbers, likely reflecting denervation–reinnervation lag and the susceptibility of target muscles to ischemia and inflammation. When multiple rejection episodes were allowed to occur and were repeatedly rescued, axon density and conduction velocity at 90 days remained comparable to controls, yet animals developed marked muscle atrophy, fibrosis, reduced contractile force, and abnormal gait (Unadkat et al. 2013). These findings indicate that repeated rejection impairs functional outcomes primarily via muscle and soft‐tissue damage rather than direct axonal loss.

In contrast, chronic rejection (CR) exerts a progressive and often irreversible impact on nerve repair, driven mainly by graft vasculopathy and fibro‐inflammatory remodeling. Human and preclinical studies consistently report neointimal hyperplasia, luminal narrowing or occlusion, dermal sclerosis, adnexal atrophy, and muscle fibrosis (Kanitakis et al. 2016; Mundinger et al. 2013; Ng et al. 2019)—all of which compromise nerve and target tissue perfusion, promote Schwann cell loss or dysfunction, and hinder remyelination. Contemporary reviews emphasize that CR is now the dominant late failure mode in VCA and involves multi‐tissue pathology with vasculopathy as a central lesion (Ng et al. 2019; Brandacher et al. 2012; Kollar et al. 2019). Experimental models of chronic rejection show macrophage and lymphocyte infiltration, upregulation of CXCL9–11, and muscle fibrosis, collectively sustaining a hypoperfused, pro‐inflammatory environment that impairs axonal regeneration and muscle reinnervation (Shah et al. 2023; Puscz et al. 2020). From a clinical perspective, chronic rejection in VCA is closely linked to diminished nerve regeneration and poorer functional outcomes, primarily through vasculopathy‐induced ischemia and fibrotic remodeling of both nerves and their target tissues (Kanitakis et al. 2016; Mundinger et al. 2013; Ng et al. 2019; Brandacher et al. 2025). While early identification and intervention aimed at limiting vasculopathy and humoral activation remain critical, current immunosuppressive regimens offer little benefit once chronic rejection is established (Ng et al. 2019; Brandacher et al. 2025). In addition, nerve repair in this setting may further amplify cross‐tissue inflammation, exacerbating the already hostile microenvironment (Shah et al. 2023).

## 4 Discussion

The findings summarized above indicate that optimizing nerve regeneration remains one of the most critical challenges for achieving functional recovery after VCA. The specific context of VCA—characterized by long nerve gaps, multiple nerve types (motor, sensory, mixed), ischemia–reperfusion injury, and chronic immunosuppression—poses unique barriers to axonal regeneration compared with conventional nerve repair. Several experimental and emerging strategies are under investigation, ranging from stem cell‐based therapies (Rau et al. 2021; Faroni et al. 2014; Khalifian et al. 2015; Panagopoulos et al. 2023; Pappalardo et al. 2019; Ching et al. 2018; Zhang and Rosen 2018) to advanced biomaterials (Long et al. 2021; Zheng et al. 2023; Fowler et al. 2014; Mukhatyar et al. 2011), targeted neurotrophic delivery (Allen et al. 2013; Xia and Lv 2018; Lopes et al. 2017; Vögelin et al. 2006; Omura et al. 2005), immunomodulatory regimens (Cilingir‐Kaya et al. 2021; Gold et al. 1995; Jost et al. 2000; Lee et al. 2000; Doolabh and Mackinnon 1999), and neuromuscular stimulation (Juckett et al. 2022; Gordon and English 2016; Gordon 2024). While many of these approaches have shown promise in standard peripheral nerve injury models, their validation in VCA remains limited, with most data coming from small animal allotransplantation studies or isolated clinical cases.

### 4.1 Cellular Therapies

Adipose‐derived stem cells (ADSCs) have emerged as a promising tool in peripheral nerve regeneration (Rau et al. 2021) due to their ability to differentiate into Schwann cell‐like cells, secrete neurotrophic factors (NGF, BDNF, GDNF, NT4) (Reid et al. 2011), and exert potent immunomodulatory effects (Pappalardo et al. 2019). Studies have demonstrated that transplanting ADSCs into nerve injury sites can enhance axonal regeneration and improve functional recovery (Faroni et al. 2014; Khalifian et al. 2015; Panagopoulos et al. 2023; Pappalardo et al. 2019). Preclinical research has shown that ADSCs can enhance nerve regeneration and improve functional outcomes in VCA (Leto Barone et al. 2015). A meta‐analysis of 44 animal studies demonstrated that incorporating ADSCs into nerve grafts significantly enhances nerve regeneration in sciatic, median, ulnar, and radial nerve lesion models across rats, dogs, monkeys, and mice (Hundepool et al. 2014). In a rat hindlimb allotransplantation model, Zhang et al. demonstrated that local administration of donor‐derived ADSCs into the graft—combined with a short course of tacrolimus—resulted in significantly prolonged graft survival (> 130 days in 85.7% of recipients) compared to systemic or contralateral delivery (Zhang et al. 2024). Local ASC treatment induced donor‐specific tolerance, as evidenced by permanent acceptance of secondary donor skin grafts without additional immunosuppression, and was associated with increased donor cell chimerism, expansion of regulatory T cells, and a shift toward protolerogenic Th2/Tr1 responses with reduction of Th1/Th17 subsets. Similarly, Chen et al. reported that ex vivo perfusion of VCA grafts with ADSCs or stromal vascular fraction (SVF) prior to transplantation prolonged graft survival and reduced histological rejection scores in a rat hindlimb model (Chen et al. 2021). Although neither study directly evaluated axonal regeneration, these findings indicate that ADSCs can shape a graft microenvironment that is less hostile to nerve regeneration by dampening alloimmune responses and sustaining local neurotrophic support. Given their dual neurotrophic and immunomodulatory properties, ADSCs represent a promising adjunct to surgical nerve repair in VCA (Knoedler et al. 2024). However, clinical translation will require studies specifically assessing their impact on functional nerve recovery in human recipients.

### 4.2 Nerve Allografts and Vascularized Nerve Allografts

Peripheral nerve reconstruction in VCA can be achieved using direct coaptation, nerve autografts, nerve allografts, or vascularized nerve allografts (VNAs) (Kornfeld et al. 2021; Grosu‐Bularda et al. 2025). While nerve autografts remain the gold standard in conventional peripheral nerve surgery, their use in VCA is limited by donor site morbidity and the already extensive reconstructive demands of the transplant procedure.

#### 4.2.1 Nerve Allografts

Conventional (non‐vascularized) nerve allografts provide structural guidance for axonal regeneration and are increasingly available in decellularized, processed forms to reduce immunogenicity (Kornfeld et al. 2021). In the setting of VCA, their use is constrained by the long regeneration distances, the mixed sensory–motor composition of major recipient nerves, and the immunogenic environment of the graft. Although systemic immunosuppression is already required for VCA, chronic immune‐mediated injury to the nerve segment may still occur, potentially compromising regeneration. Experimental allotransplantation models have shown that nerve allografts, when combined with adequate immunosuppression, can support axonal regeneration over gaps exceeding the length achievable with autografts (Broeren et al. 2024; Lans et al. 2023; Bittner et al. 2022). However, regeneration rates remain slower than in isografts, and long‐term functional outcomes are variable (Kornfeld et al. 2021; Broeren et al. 2024; Lans et al. 2023; Bittner et al. 2022).

#### 4.2.2 Vascularized Nerve Allografts

VNAs are harvested with their intrinsic blood supply and revascularized at the recipient site, which may improve axonal regeneration by preserving the viability of Schwann cells and the endoneurial microenvironment (Broeren et al. 2021; Pereira et al. 2023; Boyce et al. 2024). This vascularization can shorten ischemia time, reduce intraneural fibrosis, and improve early axonal sprouting compared to non‐vascularized grafts. In preclinical models, VNAs have shown superior histomorphometric parameters and earlier functional recovery compared to conventional allografts under identical immunosuppressive protocols (Boyce et al. 2024). Their potential advantage in VCA lies in combining immediate revascularization with the systemic immunosuppression already in place for the composite graft (Boyce et al. 2024). Nevertheless, VNAs are technically demanding, increase operative time, and require precise microsurgical anastomoses to maintain perfusion.

#### 4.2.3 Implications for VCA

Given that VCA recipients are already immunosuppressed, both nerve allografts and VNAs are theoretically viable options for reconstructing large nerve gaps when direct repair is not possible (Iske et al. 2019). VNAs may be particularly beneficial for proximal transplants or cases involving ischemic nerve segments. However, robust comparative data in VCA‐specific settings are lacking. Future studies should directly evaluate functional and histological outcomes between autografts, non‐vascularized allografts, and VNAs in clinically relevant VCA models, as well as assess the integration of adjuncts such as local neurotrophic delivery or cellular therapies.

### 4.3 Surgical and Biomaterial‐Based Approaches

In VCA, nerve repair is currently performed using direct coaptation or nerve grafts (Fowler et al. 2014), with nerve guidance conduits (NGCs) rarely employed (Table 1). Although NGCs made from collagen, chitosan, or synthetic polymers have shown robust axonal growth in conventional injury models (Long et al. 2021; Zheng et al. 2023), no published human or animal VCA study has directly compared NGCs to standard microsurgical repair. The long regeneration distances and mixed nerve composition in VCA present additional design challenges for conduits, particularly regarding luminal fillers aimed at guiding regenerating axons toward correct targets. Beyond commonly explored materials such as hydrogels, nanofibers, and membranes, preclinical studies have also investigated collagen‐based matrices which provide structural support, promote cell infiltration, and facilitate axonal alignment across the conduit lumen (Meyer, Stenberg, et al. 2016; Meyer, Wrobel, et al. 2016; Guo et al. 2018; Zor et al. 2014; Chen et al. 2006). Future work should determine whether combining NGCs with local delivery of neurotrophic or immunomodulatory agents could accelerate functional recovery in VCA without increasing operative complexity.

**TABLE 1:** Comparison of different nerve guidance conduits (NGCs) for peripheral nerve regeneration: strengths and limitations.

NGC type	Strengths	Limitations
Hollow NGCs (Grosu‐Bularda et al. 2025; Carvalho et al. 2020)	‐ FDA‐approved, clinically acceptable ‐ Prevents fibroblast infiltration ‐ Allows neurotrophic factor accumulation ‐ Prevents neuroma formation and scarring	‐ Incomplete nerve regeneration due to axonal dispersion ‐ Limited innervation of regenerated nerves
Multi‐channel NGCs (Carvalho et al. 2020)	‐ Provides topographical guidance ‐ Restricts axonal dispersion ‐ Promotes axonal regeneration ‐ Comparable to autografts in functional recovery	‐ Complex preparation methods ‐ Requires precise design for effective regeneration
Grooved NGCs (Carvalho et al. 2020)	‐ Provides directional growth cues ‐ Facilitates axon alignment and migration ‐ Can improve nerve regeneration compared to simple conduits	‐ May require additional biochemical signals for optimal results
Fiber‐filling NGCs (Carvalho et al. 2020)	‐ Provides physical and biological support ‐ Enhances cell growth and extension ‐ Ideal for long‐gap repair	‐ Requires precise control over fiber alignment and filling material ‐ May complicate preparation and application
NGCs with Conductive Fillers (Thakkar et al. 2025; Qian et al. 2019)	‐ Electrical conductivity promotes nerve regeneration ‐ Facilitates bioelectric signaling ‐ Supports neuronal growth and differentiation	‐ Potential for bioelectric interference if not properly designed ‐ Requires specific materials for conductivity
NGCs with Neurotrophic Factors (Smoliński et al. 2025; Jones et al. 2016)	‐ Promotes neuronal survival and axonal regeneration ‐ Enhances Schwann cell migration ‐ Potentially superior to autografts	‐ Release control may be difficult ‐ Short‐term release may limit long‐term nerve repair
NGCs with Support Cells (Smoliński et al. 2025; Jones et al. 2016; Faroni et al. 2015)	‐ Schwann cells (SC) and stem cells enhance regeneration ‐ SC‐based NGCs are ideal for peripheral nerve repair ‐ Stem cells promote regeneration and functional recovery	‐ Limited supply of SCs and stem cells ‐ Immune rejection and culture cycle issues ‐ Complex stem cell handling and application

### 4.4 Neurotrophic Factors

The local delivery of neurotrophic molecules—such as NGF, BDNF, GDNF, and IGF‐1—has consistently improved axonal sprouting and remyelination in peripheral nerve models (Kostereva, Wang, Fletcher, Unadkat, et al. 2016; Xia and Lv 2018; Yu et al. 2016; Mamet et al. 2003; Tajdaran et al. 2016; Tajdaran et al. 2019). Experimental data suggest that insulin‐like growth factor 1 (IGF‐1), alone or in combination with chondroitinase ABC, improves nerve regeneration following VCA (Kostereva, Wang, Fletcher, Unadkat, et al. 2016). In an allogeneic rat hind limb model maintained on low‐dose FK506 therapy, the administration of IGF‐1 and chondroitinase ABC, either alone or in combination, led to improved nerve and muscle histomorphometry compared to controls. Notably, the IGF‐1 group exhibited superior distal regeneration, as confirmed by Schwann cell immunohistochemistry, along partial extrafascicular regeneration. These findings suggest that targeted neurotrophic delivery could be integrated into VCA surgical protocols to counteract the delayed regeneration associated with long nerve gaps. However, achieving controlled, sustained release in a complex composite graft remains technically challenging, and no human application has yet been reported.

### 4.5 Immunosuppressive Therapies With Neuroregenerative Potential

Tacrolimus (FK‐506) remains the cornerstone of VCA immunosuppression and is one of the few agents with demonstrated neuroregenerative properties (Panagopoulos et al. 2023; Cilingir‐Kaya et al. 2021; Gold et al. 1995; Jost et al. 2000; Lee et al. 2000; Doolabh and Mackinnon 1999). Tacrolimus has been shown to enhance axonal regeneration in both preclinical studies and clinical case reports. A study using a murine VCA model with transgenic mice expressing fluorescent proteins in axons (Thy1‐YFP) and Schwann cells (S100‐GFP) investigated the role of FK‐506 in nerve regeneration (Yan et al. 2016). The study employed four groups of mice undergoing orthotopic limb transplantation with incomplete FK506 immunosuppression to assess nerve regeneration and Schwann cell migration. Survival and complication rates were recorded to evaluate tissue rejection. Serial imaging of axonal progression and Schwann cell viability confirmed robust nerve regeneration despite delayed rejection of skin, muscle, tendon, and bone in the transplanted limb. Histomorphometry revealed that total myelinated axon numbers at 8 weeks were comparable across all VCA groups and were not significantly different from syngeneic donor controls. Similarly, a study using a rat sciatic nerve model demonstrated that the immunosuppressive drug tacrolimus significantly enhanced motor recovery after allograft reconstruction (Kim et al. 2018). Eighty‐eight Lewis rats underwent a 1‐cm sciatic nerve allograft transplantation and skin graft from Brown‐Norway rats, comparing different immunosuppressive protocols. The results showed that all immunosuppressed groups exhibited significantly improved functional recovery compared to the control group, with no significant difference between FK506 monotherapy and combination therapy with mycophenolate mofetil (MMF). However, triple therapy (high‐dose FK506, MMF, and prednisone) was superior in reducing ankle contracture and improving electrophysiological outcomes while also effectively preventing skin graft rejection. Another study compared the efficacy of allografting versus nerve conduit implantation in a rat model of sciatic nerve regeneration (Rustemeyer and Dicke 2010). Thirty rats from two strains (Lewis and Dark Agouti) were used, with unoperated animals serving as controls. Following a 15‐mm sciatic nerve resection, one group received collagen type I conduits, while another received allografts from the other rat strain with systemic low‐dose FK506 administration. Functional recovery was assessed through walking track analysis over 16 weeks, and myelin basic protein staining was performed. The results demonstrated significantly better functional recovery in the allograft group compared to the conduit group, despite no significant difference in remyelination extent. Neither group achieved the functional or histomorphometric values of control animals. This study emphasizes the importance of systemic administration of neurotrophic molecules, such as FK506, in enhancing nerve regeneration outcomes following allograft transplantation. However, its systemic administration is linked to significant adverse effects, which limits its broader use in peripheral nerve surgeries. To mitigate this issue, researchers have developed biocompatible, ready‐to‐use delivery systems that release tacrolimus directly at the site of nerve injury (Tajdaran et al. 2016). These localized delivery devices have proven effective in promoting axonal regeneration while reducing systemic exposure to other organs (Kasper et al. 2020). A low dose of FK‐506, when combined with other immunomodulatory strategies—such as anti‐CD‐40 ligand/costimulatory blockade (Brenner et al. 2005) and cold preservation (Grand et al. 2002)—was found to promote peripheral nerve regeneration. Brenner et al. demonstrated that combining low‐dose FK‐506 (0.5 mg/kg/day) with anti‐CD40L monoclonal antibody (mAb) therapy in peripheral nerve allotransplantation showed significantly improved nerve regeneration and effective immunosuppression, with results comparable to the isograft and high‐dose FK‐506 allograft groups (Brenner et al. 2005). In fact, mice that received low‐dose FK‐506 with anti‐CD40L‐mAb demonstrated increased nerve density and fiber counts compared to the group receiving anti‐CD40L‐mAb alone, while both strategies were effective in suppressing alloreactivity. This combination was also less aggressive to nerve regeneration than high‐dose FK‐506 or high‐dose cyclosporine, both of which impaired nerve regeneration and reduced nerve fiber counts. Furthermore, this strategy ensured effective immunosuppression, as indicated by the downregulation of cytokine secretion. Conversely, administering immunosuppressive doses of FK‐506 alongside therapeutic levels of anti‐CD‐40 ligand/costimulatory blockade compromised nerve regeneration, demonstrating the delicate balance required when combining immunosuppressive treatments for optimal outcomes. A study by Grand et al. demonstrated that the combination of therapeutic doses of FK‐506 with cold preservation of nerve allografts resulted in accelerated functional recovery and superior histomorphometric outcomes compared to other groups (Grand et al. 2002). Animals treated with this combination exhibited significantly improved nerve density, reduced neural debris, and better axonal regeneration, with histomorphometric parameters that were even better than the isograft group. These results suggest that combining FK‐506 with cold preservation enhances nerve regeneration across peripheral nerve lesions, making it a promising approach for improving outcomes in nerve allotransplantation.

### 4.6 Neuromuscular Electrical Stimulation

Postoperative neuromuscular electrical stimulation (NMES) has recently been incorporated into rehabilitation protocols for face transplantation recipients, improving facial symmetry, expression range, and electromyographic patterns (Juckett et al. 2022; Gordon and English 2016; Gordon 2024; Kumar et al. 2016). Recent studies in face transplantation patients have further highlighted the potential of functional electrical stimulation (FES) in neuromuscular rehabilitation. For instance, a personalized rehabilitation program incorporating FES and cognitive methods significantly improved facial symmetry and the ability to perform basic emotional expressions in full‐face transplantation patients (Topçu et al. 2018). Surface electromyography data revealed increased similarity in facial expression patterns between patients and healthy individuals after rehabilitation, demonstrating the effectiveness of FES in enhancing motor and functional recovery (Topçu et al. 2018). These early clinical observations suggest that NMES could complement nerve regeneration in VCA by maintaining muscle viability during prolonged reinnervation and guiding functional cortical remapping. While evidence in limb transplantation is lacking, its non‐invasive nature makes it a feasible adjunct in most VCA rehabilitation programs.

**FIGURE 1:**
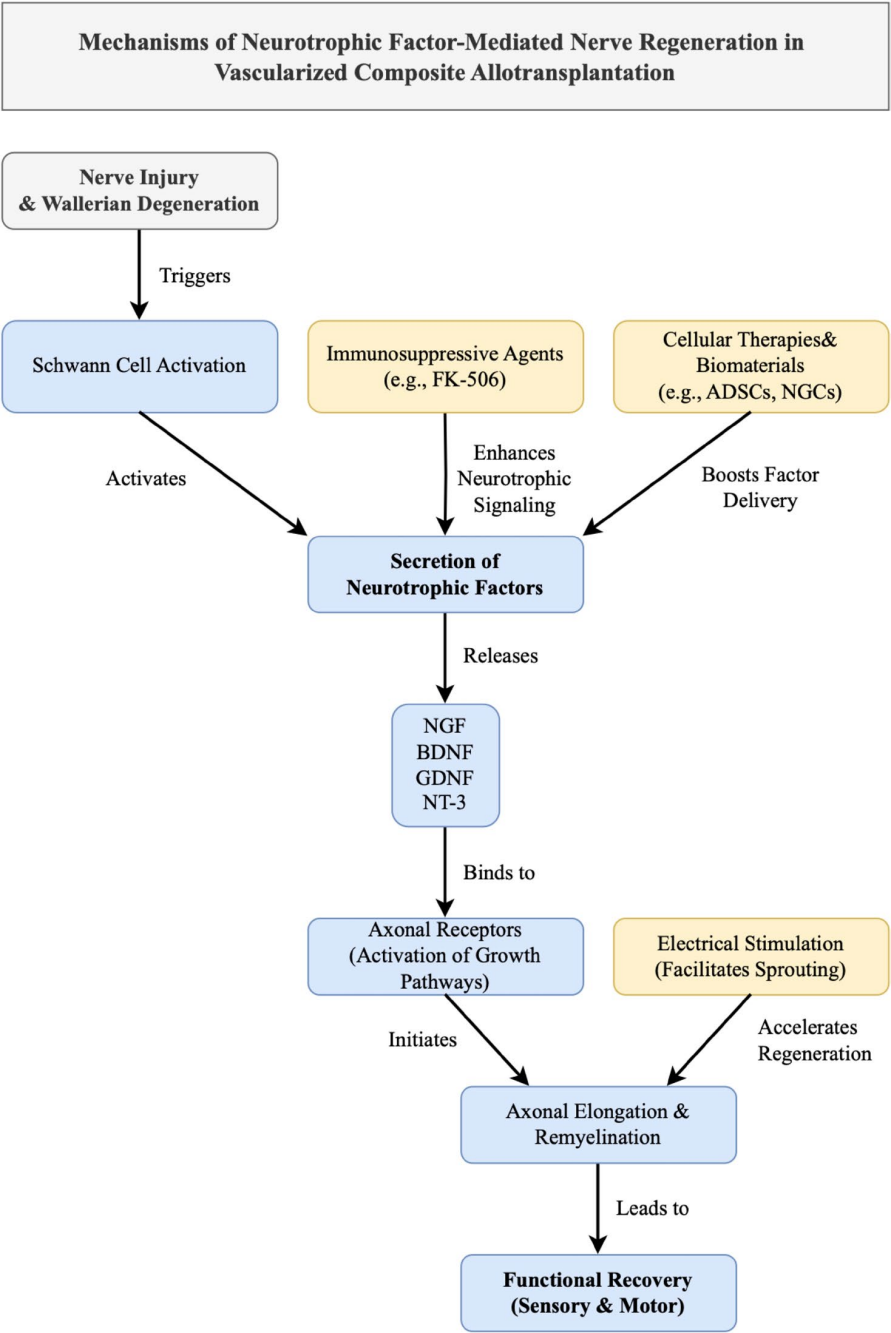
Mechanisms of neurotrophic factor‐mediated nerve regeneration in vascularized composite allotransplantation.

More comprehensive information is displayed in both Table 2 and Figure 1, illustrating how neurotrophic signaling, cellular therapies, immunosuppression, and electrical stimulation converge to promote axonal elongation and functional recovery.

**TABLE 2:** Overview of approaches for nerve regeneration and functional recovery after vascularized composite allotransplantation.

Approach	Strengths	Limitations
Cellular therapies (e.g., ADSCs) (Rhode et al. 2021; Khalifian et al. 2015; Babu et al. 2023)	‐ Promote nerve regeneration and myelination ‐ Secretion of neurotrophic factors (NGF, BDNF, GDNF) ‐ Potential to enhance the healing of nerve grafts	‐ Tumorigenic potential ‐ Variability in clinical outcomes ‐ Standardization challenges
Surgical Techniques (e.g., Nerve Grafts, Nerve Conduits) (Grosu‐Bularda et al. 2025; Malekzadeh et al. 2025; Tabatabai et al. 2025; Raza et al. 2020; Zou et al. 2024)	‐ Provide structural support for nerve regeneration ‐ Conduits can be bioengineered to enhance nerve growth	‐ Limited success for long‐gap nerve defects ‐ Autografts have donor site morbidity ‐ Conduits may not perform as well as autografts
Neurotrophic Factors (e.g., NGF, BDNF, GDNF) (Smoliński et al. 2025; Rhode et al. 2021; Kostereva, Wang, Fletcher, et al. 2016; Hoyng et al. 2014)	‐ Support axonal growth and neuronal survival ‐ Can be delivered via scaffolds for sustained release	‐ Short half‐life ‐ High doses may induce neuropathic pain
Immunosuppressive Therapies (e.g., FK‐506, Anti‐CD40L mAb) (Kostereva, Wang, Fletcher, Unadkat, et al. 2016; Daeschler et al. 2023; Yan et al. 2013)	‐ Prevents allograft rejection ‐ Improves nerve regeneration in combination with nerve grafts	‐ Increased infection risk with systemic immunosuppression ‐ Immunosuppressive regimens may not be optimal for long‐term use
Electrostimulation (Kubiak et al. 2018; Zuo et al. 2020)	‐ Enhances nerve regeneration through electrical signals ‐ Can be combined with other therapies (e.g., nerve grafts, neurotrophic factors) ‐ Improves functional recovery by stimulating axonal growth	‐ Requires careful monitoring to avoid adverse effects ‐ Efficacy can vary depending on the stimulation parameters (e.g., frequency, intensity)

Abbreviations: ADSCs, adipose tissue derived stem cells; BDNF, brain‐derived neurotropic factor; FK‐506 CD, cluster of differentiation; GDNF, glial cell‐line‐derived neurotrophic factor; mAB, monoclonal antibody; NGF, nerve growth factor.

Most strategies currently under investigation for enhancing nerve regeneration in VCA derive from broader peripheral nerve research, with only a subset tested in relevant preclinical allotransplantation models and very few applied clinically. Moving forward, research should focus on integrating these approaches into VCA‐specific protocols, accounting for the dual challenge of promoting axonal growth while maintaining immunological control. Well‐designed translational studies are needed to determine optimal combinations—such as local ADSC delivery with immunosuppressive modulation or NGCs incorporating neurotrophic release—that can realistically improve sensory and motor outcomes for VCA recipients.

## 5 Conclusion

Despite significant advancements in VCA, nerve regeneration remains a major challenge to promote functional recovery. Clinical outcomes vary widely, influenced by factors such as transplant level, rehabilitation protocols, and immunosuppression strategies. Emerging strategies, including stem cell therapies, nerve guidance conduits, and controlled neurotrophic factor delivery, show promise in enhancing nerve regeneration and functional recovery in preclinical models and other reconstructive scenarios. However, they have yet to be investigated in human VCA trials. Furthermore, standardized protocols and long‐term clinical studies are needed to refine these approaches and improve patient outcomes. Future research should focus on optimizing biomaterials, minimizing immunosuppressive requirements, and developing personalized rehabilitation strategies to maximize functional restoration in VCA recipients.

## Author Contributions

**Olivier Mathieu**, **Leonard Knoedler**, **Alexandre G. Lellouch**, **Curtis L. Cetrulo Jr.:** conceptualization. **Olivier Mathieu**, **Leonard Knoedler**, **Thomas Schaschinger**, **Jakob Fenske**, **Maxime Jeljeli**, **Alexandre G. Lellouch:** methodology. **Thomas Schaschinger**, **Jakob Fenske**, **Leonard Knoedler**, **Olivier Mathieu**, **Felix J. Klimitz**, **Maxime Jeljeli:** literature search and investigation. **Thomas Schaschinger**, **Jakob Fenske**, **Leonard Knoedler**, **Olivier Mathieu:** data curation. **Thomas Schaschinger**, **Jakob Fenske**, **Leonard Knoedler**, **Olivier Mathieu**, **Felix J. Klimitz:** formal analysis. **Thomas Schaschinger**, **Jakob Fenske**, **Leonard Knoedler:** visualization. **Leonard Knoedler**, **Olivier Mathieu:** writing – original draft. **Olivier Mathieu**, **Leonard Knoedler**, **Thomas Schaschinger**, **Jakob Fenske**, **Felix J. Klimitz**, **Maxime Jeljeli**, **Curtis L. Cetrulo Jr.**, **Alexandre G. Lellouch:** writing – review and editing. **Alexandre G. Lellouch**, **Curtis L. Cetrulo Jr.:** supervision. **Leonard Knoedler**, **Alexandre G. Lellouch:** project administration.

## Funding

The authors have nothing to report.

## Ethics Statement

The authors have nothing to report.

## Conflicts of Interest

The authors declare no conflicts of interest.

## Data Availability

The data that support the findings of this study are available from the corresponding author upon reasonable request.
